# Source of the Sun’s Heat

**Published:** 1885-02

**Authors:** 


### SOURCE OF THE SUN'S HEAT.

Prof. Ball says, in a paper recently published in the *Contemporary Review*, that the sun must have some source of heat in addition to that which it would possess by virtue of its temperature as an incandescent body. If we suppose the sun to be a vast incandescent body, formed of materials which possess the same specific heat as the materials of which our earth is composed, the sun would then cool at the rate of 5 to 10 degrees per annum. At this rate the sun could not have lasted more than a few thousand years before it cooled down. We are, therefore, compelled to inquire whether the sun may not have some other source of heat to supply its radiation beyond that which arises merely from the temperature. Of the various sources which have been suggested, it will here only be necessary to mention two. It has been supposed that the heat of the sun may be recruited by the incessant falling of the meteoric matter upon the sun's surface. If that matter had been drawn only by the sun's attraction from the remote depths of space, it would fall upon the sun with an enormously great velocity, amounting to about 300 miles a second. It follows from the principal of the equivalence between heat and mechanical energy, that a body entering the sun with this velocity would contribute to the sun a considerable quantity of heat. It is known that small meteoroids abound in the solar system; they are constantly seen in the form of shooting stars when they dash into our atmosphere, and it can hardly be doubted that myriads of such bodies must fall into the sun. It does not, however, seem likely that enough matter of this kind can enter the sun to account for its mighty radiation of heat. It can be shown that the quantity of matter necessary for this purpose is so large, that a mass equal in the aggregate to the mass of the earth, would have to fall into the sun every century, if the radiation of the sun were to be defrayed from this source. That so large a stream of matter should be perenially drawn into the sun is, to say the least, highly improbable. But it is quite possible to account for the radiation of the sun on strictly scientific principles, even if we discard entirely the contributions due to meteoric matter. As the sun parts with its heat it must contract, in virtue of the general law that all bodies contract when cooling; but in the act of contraction an amount of the heat is produced. By this the process of cooling is greatly retarded. It can, indeed, be shown that if the sun contracts so that his diameter decreases one mile every twenty-five years, the amount of heat necessary to supply his radiation would be amply accounted for. At this rate many thousands of years must elapse before the diminution of the sun's diameter would be large enough to be appreciable by our measurements.

